# Engineering of anti-human interleukin-4 receptor alpha antibodies with potent antagonistic activity

**DOI:** 10.1038/s41598-019-44253-9

**Published:** 2019-05-23

**Authors:** Jung-Eun Kim, Keunok Jung, Jeong-Ah Kim, Seung-Hyun Kim, Hae-Sim Park, Yong-Sung Kim

**Affiliations:** 1 Department of Molecular Science and Technology, Ajou University, Suwon, 16499 Republic of Korea; 2 Department of Allergy and Clinical Immunology, Ajou University School of Medicine, Suwon, 16499 Republic of Korea

**Keywords:** Antibody therapy, Interleukins

## Abstract

Development of antagonistic antibody (Ab) against interleukin-4 receptor alpha (IL-4Rα) subunit of IL-4/IL-13 receptors is a promising therapeutic strategy for T helper 2 (T_H_2)-mediated allergic diseases such as asthma and atopic dermatitis. Here we isolated anti-human IL-4Rα antagonistic Abs from a large yeast surface-displayed human Ab library and further engineered their complementarity-determining regions to improve the affinity using yeast display technology, finally generating a candidate Ab, 4R34.1.19. When reformatted as human IgG1 form, 4R34.1.19 specifically bound to IL-4Rα with a high affinity (*K*_D_ ≈ 178 pM) and effectively blocked IL-4- and IL-13-dependent signaling in a reporter cell system at a comparable level to that of the clinically approved anti-IL-4Rα dupilumab Ab analogue. Epitope mapping by alanine scanning mutagenesis revealed that 4R34.1.19 mainly bound to IL-4 binding sites on IL-4Rα with different epitopes from those of dupilumab analogue. Further, 4R34.1.19 efficiently inhibited IL-4-dependent proliferation of T cells among human peripheral blood mononuclear cells and suppressed the differentiation of naïve CD4^+^ T cells from healthy donors and asthmatic patients into T_H_2 cells, the activities of which were comparable to those of dupilumab analogue. Our work demonstrates that both affinity and epitope are critical factors for the efficacy of anti-IL-4Rα antagonistic Abs.

## Introduction

Allergic diseases such as asthma and atopic dermatitis affect a huge population globally, but a subset of severe cases are not managed efficiently^1^. Allergic diseases are mainly driven by type 2 inflammation encompassing CD4^+^ T helper 2 (T_H_2) cell-mediated responses^2^. The type 2 inflammation is characterized by the overproduction of type 2 cytokines (e.g. Interleukin (IL)-4, IL-13 and IL-5), which mediate activation of immune cells including eosinophils and mast cells as well as isotype switching of B cells to IgE production^3^. Rather than systemically immunosuppressing chemical agents, biologics, including antibodies (Abs), targeting a specific type 2 cytokine or the receptor has emerged as a promising therapeutic strategy because it shows substantial therapeutic benefits in patients with severe asthma and atopic dermatitis^1,4,5^.

IL-4/IL-13 signaling is a very attractive target to inhibit type 2 inflammation because it drives the differentiation and clonal expansion of naïve CD4^+^ T cells into T_H_2 cells and further production of cytokines IL-4, IL-5 and IL-13 from T_H_2 cells in the upstream pathways. This subsequently leads to the downstream pathways of Immunoglobulin (Ig) E production, eosinophil activation, and mucus secretion^3,6^. IL-4 can signal through two types of heterodimeric receptor complexes: type I receptor composed of IL-4 receptor alpha subunit (IL-4Rα) and cytokine receptor common γ-chain (γc) and type II receptor composed of IL-4Rα and IL-13 receptor alpha 1 subunit (IL-13Rα1)^7^. However, IL-13 signals only via the type II receptor^8^. Type I receptor is mainly expressed on the surface of hematopoietic immune cells, whereas type II receptor is found on non-hematopoietic cells^7^.

To block either IL-4 or IL-13 signaling pathway, Abs targeting the individual cytokine IL-4 or IL-13 have been developed^9^. However, these Abs have not shown any favorable therapeutic benefits in clinical trials for asthma, suggesting that blocking IL-4 or IL-13 alone might be insufficient because of the redundancy in their signaling pathways. Because the activation of IL-4Rα is utilized by both IL-4 and IL-13, it provides a single target to block signaling of both cytokines. For example, dupilumab (Dupixent^TM^), a humanized IgG4 Ab directed against human IL-4Rα, has been approved by U.S. Food & Drug Administration for the treatment of atopic dermatitis^10,11^ and is now under clinical trials for treating asthma, suggesting that IL-4/IL-13 dual blocking by IL-4Rα antagonist is a plausible way for treating allergic diseases^12^. Another anti-IL-4Rα antagonistic IL-4 mutant protein, pitrakinra, is now being evaluated for asthma^13^. However, AMG 317, a human IgG2 Ab against IL-4Rα, did not demonstrate favorable clinical efficacy across patients with moderate to severe atopic asthma in phase II study^14^.

There is an urgent need for novel IL-4Rα antagonistic Abs because a large number of patients with allergic diseases still have no treatment option^3^. In this study, we performed Ab library screening and engineering to generate potent Abs against human IL-4Rα using yeast surface display technology. The finally engineered human IgG1 Ab 4R34.1.19 specifically bound to IL-4Rα with high affinity to competitively block the ligand binding to IL-4Rα and thereby suppressed both IL-4- and IL-13-dependent signaling in *ex vivo* assays with human immune cells at comparable levels of dupilumab analogue. 4R34.1.19 differed from dupilumab analogue particularly in the binding epitopes and kinetics.

## Results

### Isolation and characterization of anti-IL-4Rα Abs from yeast surface-displayed human Fab library

**Figure 1:**
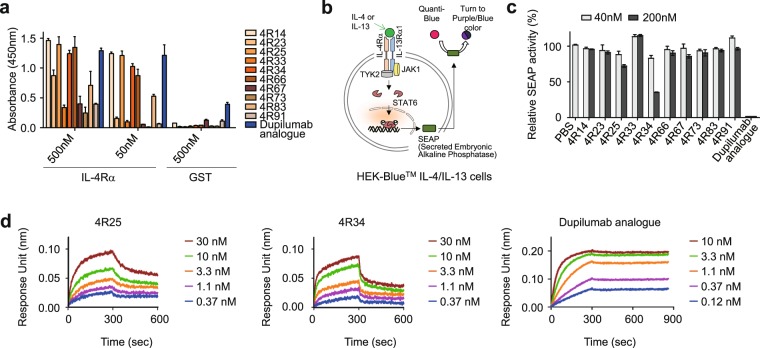
Isolation and characterization of human Abs directed against IL-4Rα. (**a**) Binding activity of the isolated anti-IL-4Rα Abs to plate-coated human IL-4Rα or GST, as determined by ELISA. Data represented as mean ± SD (n = 3). (**b**) Schematic diagram of the reporter HEK-Blue^TM^ IL-4/IL-13 cell line to monitor the biological activity of anti-IL-4Rα Abs. The details are described in the text. (**c**) IL-4Rα-blocking activity of the indicated Abs, as determined by SEAP secretion levels from HEK-Blue^TM^ IL-4/IL-13 cells after stimulation with rhIL-4 (100 pM) in the presence of the Abs (40 and 200 nM) for 24 h. Data are presented as percentage (mean ± SD (n = 3)) in SEAP levels relative to PBS-treated samples. (**d**) Binding isotherms of the immobilized anti-IL-4Rα Abs to soluble antigen IL-4Rα, measured by bio‐layer interferometry on OctetRED96 (Fortebio). The concentrations of IL-4Rα analysed are indicated (colored).

The extracellular domain of human IL-4Rα (residues 26–232) contains a cytokine binding region and six potential N-glycosylation sites^15^. For the soluble antigen preparation, the ectodomain of IL-4Rα with C-terminus 6× His tag was expressed in mammalian human embryonic kidney (HEK) 293 F cell cultures. The purified protein (~45 kDa) was detected as a single band (~26 kDa) after deglycosylation by PNGase F, indicative of multiple N-glycosylations in the purified protein (Supplementary Fig. 1). For Ab library, we used human synthetic Ab libraries displayed in the format of antigen binding fragment (Fab) on the surface of yeast diploid cells^16^. To isolate IL-4Rα-specific Ab, the yeast library was screened against biotinylated IL-4Rα protein by one round of magnetic activated cell sorting (MACS), followed by three rounds of fluorescence activated cell sorting (FACS) by gradually decreasing antigen concentration in every round. The sorted yeast cells were plated on the selective medium and 96 individual clones were randomly analysed to yield 10 unique high affinity binders. The 10 Fabs were converted into the conventional human IgG1 format and expressed in HEK293F cell cultures. As a positive control Ab, we used dupilumab analogue, which has identical amino acid sequences of the variable domain of heavy chain (VH) and whole light chain to the commercial product of dupilumab but has the constant region of human IgG1 isotype rather than the IgG4 isotype of dupilumab. In enzyme-linked immunosorbent assay (ELISA), the isolated Abs showed concentration-dependent binding to IL-4Rα at varying levels but not to off-target glutathione S-transferase (GST) (Fig. 1a).

We initially tested all isolated Abs for the biological activity because biological potency of Abs is often determined by epitope binding rather than binding affinity^17^. For this, we used a HEK-Blue^TM^ IL-4/IL-13 cell line, in which HEK293 cells are engineered to produce secreted embryonic alkaline phosphatase (SEAP) in the supernatant in response to IL-4 or IL-13 stimulation^18,19^. Thus, IL-4/IL-13-dependent type I receptor activation can be monitored by quantification of the enzymatic activity of SEAP using QUANTI-Blue^TM^ (Fig. 1b). Among the tested clones, 4 R25 and 4R34 showed the highest IL-4-blocking activity of 28% and 72%, respectively (Fig. 1c). However, those were much less effective than dupilumab analogue (95%), a reference Ab.

To understand the difference in the biological activities between the Abs, we determined the equilibrium dissociation constant (*K*_D_) by bio-layer interferometry. Compared to monovalent antigen binding fragment (Fab), IgG Ab with two Fab arms could exhibit the accumulated strength of binding affinity from bivalent binding interactions with antigens (so called “avidity” effect). To avoid avidity effect, Ab was first immobilized onto anti-human IgG Fc capture (AHC) biosensor at a low density and then binding kinetics was measured with soluble monomeric IL-4Rα. 4R34 showed stronger binding affinity (*K*_D_ ≈ 1.04 nM) than 4R25 (*K*_D_ ≈ 2.36 nM), but was much weaker than the binding affinity of dupilumab analogue (*K*_D_ ≈ 9.16 pM) (Fig. 1d and Table 1). Thus, the IL-4-blocking activities of Abs were in order of the affinity to IL-4Rα. Given that IL-4 binds to IL-4Rα with high affinity (*K*_D_ ≈ 20 to 300 pM)^20^, we reasoned that a higher receptor binding affinity is required for more potent antagonistic Abs. Accordingly, we chose 4 R34 for affinity maturation because it showed superior IL-4-blocking activity as compared to the IL-4-blocking activity of 4R25.

**Table 1:** Binding kinetics of the isolated IL-4Rα Abs to IL-4Rα^a^.

	IgG Abs	*k*_on_ (1/M s)	*k*_off_ (1/s)	*K*_D_ (M)	*R* ^2^
Initial screening	Dupilumab analogue	(2.99 ± 0.02) × 10^6^	(2.74 ± 0.33) × 10^−5^	(9.16 ± 1.11) × 10^−12^	0.99
	4 R25	(8.43 ± 0.16) × 10^5^	(1.99 ± 0.03) × 10^−3^	(2.36 ± 0.06) × 10^−9^	0.95
	4 R34	(3.47 ± 0.01) × 10^6^	(3.61 ± 0.05) × 10^−3^	(1.04 ± 0.04) × 10^−9^	0.90
1st affinity maturation	4R34.1	(4.28 ± 0.05) × 10^6^	(1.72 ± 0.01) × 10^−3^	(4.01 ± 0.06) × 10^−10^	0.96
	4R34.2	(4.70 ± 0.09) × 10^6^	(4.01 ± 0.02) × 10^−3^	(8.53 ± 0.02) × 10^−10^	0.94
	4R34.19	(4.58 ± 0.08) × 10^6^	(2.43 ± 0.01) × 10^−3^	(5.32 ± 0.10) × 10^−10^	0.96
	4R34.29	(5.18 ± 0.11) × 10^6^	(3.71 ± 0.03) × 10^−3^	(7.16 ± 0.16) × 10^−10^	0.95
2nd affinity maturation	4R34.1.11	(4.62 ± 0.05) × 10^6^	(9.68 ± 0.06) × 10^−4^	(2.10 ± 0.03) × 10^−10^	0.98
	4R34.1.13	(5.12 ± 0.05) × 10^6^	(1.07 ± 0.06) × 10^−3^	(2.08 ± 0.02) × 10^−10^	0.98
	4R34.1.17	(4.92 ± 0.06) × 10^6^	(8.79 ± 0.07) × 10^−4^	(1.79 ± 0.02) × 10^−10^	0.97
	4R34.1.18	(6.05 ± 0.09) × 10^6^	(1.75 ± 0.06) × 10^−3^	(2.90 ± 0.04) × 10^−10^	0.96
	4R34.1.19	(4.93 ± 0.06) × 10^6^	(8.77 ± 0.06) × 10^−4^	(1.78 ± 0.02) × 10^−10^	0.98
	4R34.1.21	(4.63 ± 0.04) × 10^6^	(1.06 ± 0.05) × 10^−3^	(2.28 ± 0.02) × 10^−10^	0.99

^a^Binding kinetic of interactions between soluble IL-4Rα and immobilized anti-IL-4Rα Abs, as measured by bio-layer interferometry. The association rate constant (*k*_on_), dissociation rate constant (*k*_off_), equilibrium dissociation constant (*K*_D_), and estimate of the goodness of the curve fit (*R*^2^) were obtained using the Octet Data Analysis software (ForteBio). IgG, immunoglobulin G.

### Affinity maturation of 4R34 Ab by complementarity-determining region (CDR) mutations

**Figure 2:**
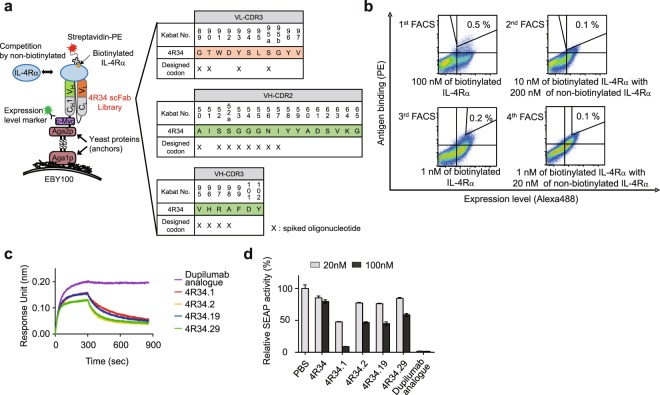
Affinity maturation of 4 R34 and characterization of the isolated clones. (**a**) Scheme of library construction and screening of 4R34 in the format of scFab using yeast surface display technology. The indicated residues in VL-CDR3, VH-CDR2, and VH-CDR3, highlighted by “X,” were randomly mutated, while maintaining the original amino acids at each residue of 4R34 at a frequency of approximately 50%, using designed spiked oligonucleotides. Numbering is according to the Kabat definition. (**b**) Flow cytometric analysis of antigen binding and expression levels of 4R34-based scFab yeast library in each round screening by FACS. The screening conditions of antigen and sorting gate used in each round are indicated. (**c**) Comparison of association and dissociation of soluble IL-4Rα antigen at 10 nM to immobilized anti-IL-4Rα antibodies (Abs), as measured by bio‐layer interferometry. (**d**) IL-4Rα-blocking activity of the indicated Abs, as determined by SEAP secretion levels from HEK-Blue^TM^ IL-4/IL-13 cells after stimulation with rhIL-4 (100 pM) in the presence of the Abs (20 and 100 nM) for 24 h. Data are presented as percentage (mean ± SD (n = 3)) in SEAP levels relative to PBS-treated samples.

Because optimization of multiple CDRs is usually required for affinity maturation to obtain additive or synergistic effects, we chose mutagenesis on multiple CDRs for the affinity maturation^21^. Previous structural analysis of many Ab–protein antigen complex structures have revealed that contribution of six CDRs of the variable domains of heavy chain (VH) and light chain (VL) to antigen binding is in order of the third CDR of VH (VH-CDR3) > VH-CDR2 > VL-CDR3 > VH-CDR1 ≈ VL-CDR1 > VL-CDR2^22^. Thus, we first chose VH-CDR3, VH-CDR2, and VL-CDR3 regions for mutagenesis in library construction. Further, to cover as much as the theoretical diversity of Ab library with yeast display technology, we focused on the solvent exposed residues, rather than all residues, of the CDRs, known to be most potentially contributing to antigen recognition^23^ as follows: VH-CDR3 (residues 95–98, Kabat numbering)^24^, VH-CDR2 (residues 50, 52–57), and VL-CDR3 (residues 89, 90, 93, and 95a) (Fig. 2a). The targeted residues were randomized using hand-mixed spiked oligonucleotides, which were designed to randomly mutate each residue while retaining the parental amino acids at a level of ~50% at each residue^25,26^, to conserve parental residues critically contributing to IL-4Rα binding (Fig. 2a). We amplified VH and VL gene libraries on 4R34 template to be combined into single-chain Fab (scFab) format with a G4S-based 63 amino-acid linker^27^, generating a library with a diversity of approximately 3.0 × 10^7^. Sequencing of tens of clones confirmed the fidelity of the library diversity.

We screened the library by FACS after alternative antigen labelling for equilibrium (low antigen concentration) and kinetics (long dissociation time) to isolate high affinity binder^28,29^. In the first and third round, equilibrium screenings were performed by incubating the library with 100 nM and 1 nM of biotinylated IL-4Rα, respectively. For the kinetic screening in second and fourth round, the scFab library labeled with 10 and 1 nM of biotinylated IL-4Rα, respectively. This was further incubated for an additional 4 h with 20-fold molar excess of non-biotinylated IL-4Rα as a competitor to allow antigen dissociation prior to sorting (Fig. 2b). After the final sorting, 47 high binding clones were randomly analysed yielding four unique clones (4 R34.1, 4R34.2, 4R34.19, and 4R34.29) (Supplementary Fig. 2a).

The isolated scFab clones were reformatted as human IgG1 form and then purified for further characterization. Affinity analysis of the clones showed the improved binding affinity ranged from *K*_D_ ≈ 401 pM to 853 pM, compared with the parental 4R34 (Table 1). However, these affinities were still much lower than that of dupilumab analogue (*K*_D_ ≈ 9.16 pM), the high affinity of which was mostly attributed to the extremely low dissociation rate (off-rate) constant (koff) (Fig. 2c and Table 1). In the functional assay with HEK-Blue^TM^ IL-4/IL-13 cells, all the Abs displayed improved IL-4-blocking activity compared with the IL-4-blocking activity of parental 4R34, but the inhibitory activity was much less than that of dupilumab analogue (Fig. 2d), prompting further affinity maturation, particularly off-rate improvement. Noticeably, though 4R34.1 Ab exhibited similar equilibrium and kinetic binding parameters to those of the other three Abs and it showed the much higher IL-4Rα antagonistic activity (Fig. 2d), suggesting that binding epitope also plays a critical role in the biological efficacy in addition to the binding affinity. Based on these results, 4R34.1 was selected for another round of affinity maturation.

### Engineering of 4R34.1 to generate more potent 4R34.1.19 Ab

**Figure 3:**
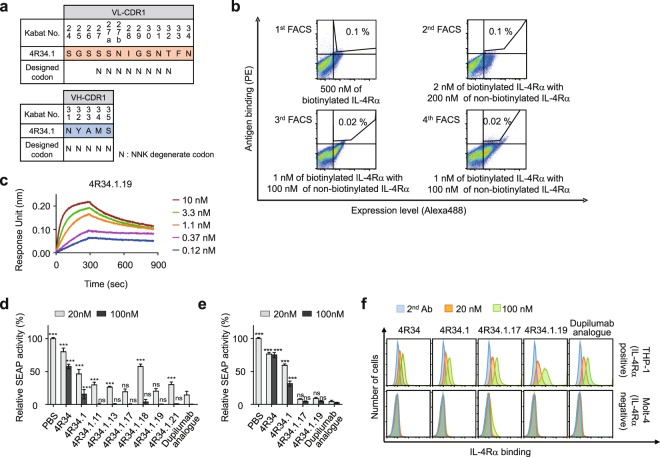
Engineering and characterization of 4 R34.1.19. (**a**) Library construction scheme of 4 R34.1, where the indicated residues in the VL-CDR1 and VH-CDR1 were randomized with NNK degenerate codon that encodes all 20 amino acids. (**b**) Flow cytometric analysis of antigen binding and expression levels of 4 R34.1-based scFab yeast library in each round screening by FACS. The screening conditions of antigen and sorting gate used in each round are indicated. (**c**) Binding isotherms of the immobilized anti-IL-4Rα Ab 4R34.1.19 to soluble antigen IL-4Rα, measured by bio‐layer interferometry. The concentrations of IL-4Rα analysed are indicated (colored). (**d**,**e**) IL-4Rα blocking activity of the indicated Abs, as determined by SEAP levels from HEK-Blue^TM^ IL-4/IL-13 cells after stimulation with rhIL-4 (100 pM) (**d**) or rhIL-13 (1 nM) (**e**) in the presence of the Abs (20 and 100 nM) for 24 h. Data are presented as percentage (mean ± SD (n = 3)) in SEAP levels relative to phosphate buffer saline (PBS)-treated samples. Statistical analysis was performed using a two-way ANOVA followed by the Newman-Keuls post-test. *P < 0.05, **P < 0.01, ***P < 0.001; ns, not significant versus dupilumab analogue. (**f**) Binding specificity of the indicated Abs (20 and 100 nM) for cell surface expressed IL-4Rα, as analysed in IL-4Rα-expressing THP-1 cells and IL-4Rα-deficient Molt-4 cells by flow cytometry. Representative histograms from three independent experiments are shown.

For further optimization of 4R34.1, the two unexplored CDRs, VH-CDR1 and VL-CDR1, were randomized focusing on the solvent-accessible positions of VL-CDR1 (residues 27–32) and VH-CDR1 (residues 31–35) with a degenerated codon of NNK, which encodes all 20 amino acids with a reduced stop codon frequency^30^ (Fig. 3a). We used the highly diversifiable NNK codon for both affinity maturation and minor changes in the epitopes (i.e. epitope drift). Because high diversities in the CDR loop might lead to loss of antigen binding ability, relatively high antigen concentration (500 nM) was used to screen the library in the first round of FACS. In the subsequent rounds 2 to 4, kinetic screening was performed by saturating the library with 1–2 nM of biotinylated IL-4Rα and then incubating with 100-fold molar excess of non-biotinylated IL-4Rα in 10 mL volume at 25 °C for 2 h for kinetic competition^29^ (Fig. 3b). After the final sorting, we analysed tens of scFab clones to yield six unique clones. The isolated clones showed dramatic diversification in sequence of VL-CDR1 region but conserved the VH-CDR1 sequence except for 4 R34.1.19 that had mutated sequence in VH-CDR1 region as well, compared with the parent 4 R34.1 (Supplementary Fig. 2b). Again, the isolated clones converted to human IgG1 form were purified for further characterization.

Kinetic binding analysis of the isolated clones revealed ~2- to 4-fold higher affinity with IL-4Rα (*K*_D_ ≈ 178 pM to 290 pM), mainly because of the slower dissociation rate constants (i.e. lower *k*_off_), compared with the parental 4 R34.1 (Fig. 3c and Table 1). Nonetheless, the affinities of the isolated Abs were still approximately 20-fold lower than the affinity of dupilumab analogue (Fig. 3c and Table 1). In the functional assay, most clones showed higher IL-4Rα-blocking activities than the parental 4R34 and 4R34.1 Abs in IL-4-stimulated HEK-Blue^TM^ IL-4/IL-13 cells (Fig. 3d). Remarkably, two Abs, 4R34.1.17 and 4R34.1.19, exhibited IL-4Rα-blocking activities comparable to that of dupilumab analogue (Fig. 3d), despite their lower binding affinity. Given that IL-4Rα is also essential in IL-13 signaling through the type I receptor (IL-4Rα/IL-13Rα1)^31^, the two best Abs were further characterized for IL-13-blocking activity in HEK-Blue^TM^ IL-4/IL-13 cells. The two clones exhibited more potent inhibitory activity of IL-13-mediated signaling than the parent 4 R34 and 4 R34.1 at a similar level to that of dupilumab analogue (Fig. 3e). These data demonstrated the very potent inhibition activity of 4 R34.1.17 and 4 R34.1.19 Abs for both IL-4- and IL-13-mediated signaling by IL-4Rα blockade, equivalent to that of dupilumab analogue.

High affinity-matured Abs often display non-specificity, which can lead to undesirable off-target effects and poor *in vivo* pharmacokinetics because of nonspecific tissue binding^32,33^. Thus, we assessed the binding specificity of the Abs for endogenous IL-4Rα expressed on cell surface using IL-4Rα-positive THP-1 cells and IL-4Rα-negative Molt-4 cells by flow cytometry. Clones of 4R34, 4R34.1, and 4R34.1.19, including dupilumab analogue, showed dose-dependent specific binding to the cell surface expressed IL-4Rα, whereas 4R34.1.17 showed nonspecific binding at a high concentration (Fig. 3f). We thus finally selected 4R34.1.19 as the final candidate Ab. We further evaluated developability of 4R34.1.19 in the aspect of thermal stability and non-specificity. During incubation at 50 °C up to 48 h, both 4 R34.1.19 and dupilumab analogue maintained selective binding to IL-4Rα without substantial formation of soluble oligomers compared with those incubated at 4 °C (Supplementary Fig. 3a,b), indicating that 4 R34.1.19 possesses comparable thermal stability to that of dupilumab analogue. We tested polyreactivity of 4 R34.1.19 Ab by multiantigen ELISA using four structurally different antigens (double-stranded DNA (dsDNA), insulin, hemocyanin, and cardiolipin^34^. 4 R39.1.19 did not bind to all antigens (Supplementary Fig. 3c), like dupilumab analogue, indicative of the absence of non-specificity of 4 R39.1.19. Taken together, the above results suggest that 4 R34.1.19 possesses favorable developability comparable to that of dupilumab analogue.

### Epitope mapping of IL-4Rα antagonistic Abs

**Figure 4:**
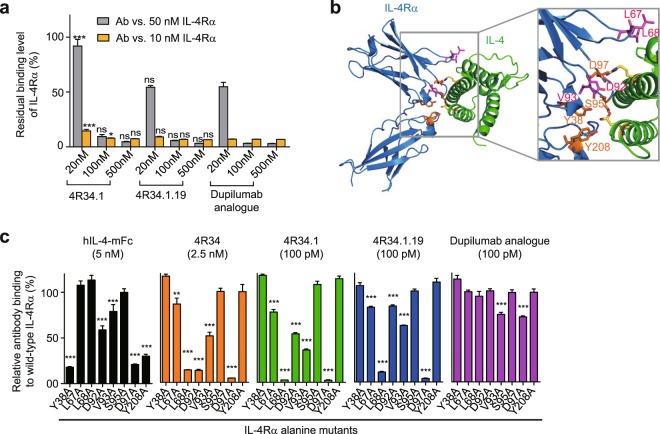
Epitope mapping of anti-IL-4Rα Abs by alanine scanning mutagenesis. (**a**) Competitive ELISA showing the percentage of bound IL-4Rα (10 and 50 nM) to plate-coated hIL-4-mFc in the presence of the indicated Abs (20, 100, and 500 nM) compared to that without the Ab competitor. Data are represented as mean ± SD (n = 3). Statistical analyses were performed using a two-way ANOVA followed by the Newman-Keuls post-test. *P < 0.05, **P < 0.01, ***P < 0.001; ns, not significant versus dupilumab analogue. (**b**) Overall structure of the human IL-4Rα:IL-4 complex (PDB: 1IAR). Magnified section shows the residues of IL-4Rα putatively involved in IL-4 binding. (**c**) The percent relative binding of the indicated hIL-4-mFc (5 nM) and anti-IL-4Rα Abs (2.5 nM of 4 R34 and 100 pM of 4 R34.1, 4 R34.1.19 and dupilumab analogue) to IL-4Rα alanine mutants compared to that of wild-type IL-4Rα. Data are represented as mean ± SD (n = 3). Statistical analyses were performed using a one-way ANOVA followed by the Newman-Keuls post-test. *P < 0.05, **P < 0.01, ***P < 0.001 versus binding to wild-type IL-4Rα.

The binding surface and amino acids of IL-4Rα to bind IL-4 are nearly identical to those of IL-13^8^. In competitive ELISA, the isolated Abs, 4 R34.1 and 4 R34.1.19, efficiently competed with human IL-4Rα for binding to human IL-4 fused to the C-terminus of mouse IgG2a Fc (hIL-4-mFc) (Fig. 4a, Supplementary Fig. 4a), like dupilumab analogue. We further sought to identify epitopes of the Abs by alanine scanning mutagenesis focusing on 8 residues of IL-4Rα, located in the interface with the ligands of IL-4/IL-13^15^ (Fig. 4b). In total 8 alanine mutants of IL-4Rα were prepared (Supplementary Fig. 4b) and subjected to ELISA for Ab binding assay. The IL-4 analogue, hIL-4-mFc, significantly lost the binding to IL-4Rα mutants carrying an alanine substitution at Y38, D92, V93, D97, and Y208 (Fig. 4c), confirming the highest energetic contribution of the residues to the ligand binding^15^. The isolated anti-IL-4Rα Abs substantially lost the binding activities with considerable varying degrees for the alanine mutants at L67, L68, D92, V93, and D97 residues (Fig. 4c, Supplementary Fig. 4c), indicating that they mainly recognize the ligand binding regions on IL-4Rα, but their epitopes are slightly different to each other. Noticeably, the binding profile of 4 R34.1.19 for the alanine mutants were much different from that of dupilumab analogue (Fig. 4c, Supplementary Fig. 4c), indicative of their different epitopes on IL-4Rα. 4 R34.1.19 significantly lost binding affinity for IL-4Rα mutants carrying an alanine substitution mainly at IL-4 binding sites (L68, V93 and D97), whereas dupilumab analogue only slightly lost binding ability to V93A and D97A mutants. Although dupilumab has been known to compete with both IL-4 and IL-13 for IL-4Rα binding (Patent US 7,605,237 B2), no information is available on the specific epitope. Taken together, these results suggest that the anti-IL-4Rα Abs recognize the ligand binding (IL-4/IL-13) sites of IL-4Rα, but different from those of dupilumab analogue, to compete with the ligand for binding to IL-4Rα.

### 4R34.1.19 inhibits IL-4-dependent T cell proliferation and T_H_2 differentiation

**Figure 5:**
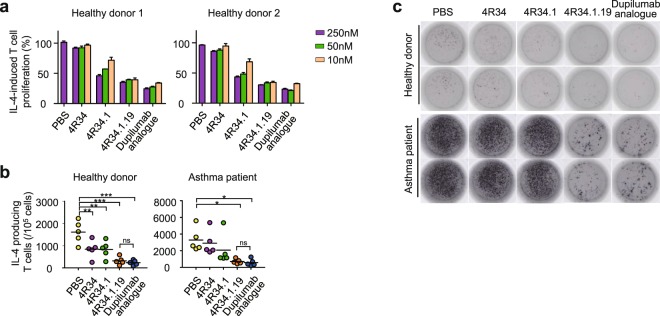
Inhibitory effects of anti-IL-4Rα Abs on IL-4-stimulated T cell proliferation and T_H_2 differentiation. (**a**) Dose-dependent blocking effects of anti-IL-4Rα Abs on the proliferation of T cells among PHA-activated PBMCs in response to rhIL-4 (500 pM), determined by CTG assay after 72 h culture. Data are represented as mean ± SD (n = 3). (**b,c**) Inhibitory effects of anti-IL-4Rα Abs (100 nM) on the T_H_2 differentiation of naïve CD4^+^CD45RO^−^T cells from healthy donors or asthmatic patients after 7 days culture in T_H_2-skewing conditions in the presence of rhIL-4 (500 pM) and anti-IL-4Rα Abs (100 nM). The number of IL-4-producing T_H_2 cells were determined by ELISPOT. Quantification of spot forming T cells (**b**) and representative image both healthy donor and asthmatic patient (**c**) are shown. In (**b**), error bars, ± SD (n = 3). Statistical analyses were performed using a one-way ANOVA followed by the Newman-Keuls post-test. *P < 0.05, **P < 0.01, ***P < 0.001; ns, not significant versus PBS-treated group.

IL-4-blocking agents have shown the ability to inhibit IL-4-dependent T cell proliferation among human peripheral blood mononuclear cells (PBMCs)^35–37^. Stimulation of human PBMCs by T cell mitogen PHA (phytohemagglutinin) enriches the population dominantly with T cells (routinely more than 90% T cells)^38^, which further proliferate in response to IL-4^35,37^. Most T cells in the PHA- and then IL-4-activated PBMC population could minimize the possible effector functions by other immune cells, such as antibody-dependent cellular cytotoxicity. 4 R34.1.19 displayed a dose-dependent blocking activity for IL-4-induced T cell proliferation in PHA-stimulated PBMCs from two healthy donors, showing greater activity than that of the parental 4 R34 and 4 R34.1 Abs, but comparable activity to that of dupilumab analogue (Fig. 5a).

Because IL-4 has been implicated in promoting T_H_2 responses by driving the differentiation of naïve CD4^+^ T cells into T_H_2 cells^3^, we next assessed the ability of 4 R34.1.19 to inhibit IL-4-induced differentiation of naïve CD4^+^ T cells into T_H_2 cells. Purified naïve CD4^+^CD45RO^− ^T cells from healthy donor PBMCs were cultured under the T_H_2-polarizing conditions including IL-4^39^ with anti-IL-4Rα Abs for 7 days. Then, the cells were transferred to ELISPOT plates to quantify the number of IL-4-secreting differentiated T_H_2 cells^40^. The anti-IL-4Rα Abs reduced the number of IL-4 secreting T_H_2 cells with the superior potency of 4 R34.1.19 over the parental clones (4 R34 and 4 R34.1) (Fig. 5b,c, Supplementary Fig. 5), indicating that they blocked the differentiation of naïve CD4^+^ T cells into T_H_2 cells. Especially, 4 R34.1.19 showed the comparable potency to that dupilumab analogue.

We further evaluated the blocking activity of anti-IL-4Rα Abs with naïve CD4^+^CD45RO^− ^T cells purified from PBMCs of asthmatic patients. We found that the patient-derived naïve CD4^+^ T cells differentiated more efficiently into T_H_2 cells in response to IL-4 than those from healthy donors (Fig. 5b,c, Supplementary Fig. 5), suggesting that the naïve CD4^+^ T cells from asthmatic patients are more prone to be polarized to T_H_2 cells^40^. Compared with the parental 4 R34 and 4 R34.1 Abs, 4 R34.1.19 substantially inhibited the IL-4-mediated differentiation of patient-derived naïve CD4^+^ T cells into T_H_2 cells at comparable levels to dupilumab analogue.

## Discussion

IL-4Rα is a well-validated target to develop antagonist Ab for blocking both IL-4- and IL-13-mediated type 2 inflammation, as evidenced by the clinically approved anti-IL-4Rα dupilumab. In this study, we isolated anti-IL-4Rα antagonistic Abs from the large synthetic human Fab libraries displayed on yeast cell surface and further engineered their CDRs to improve the affinity using yeast display technology to finally generate a potent IL-4Rα antagonistic Ab, 4 R34.1.19. The initially isolated Abs showed IL-4Rα-blocking activity; however, they were less effective than the reference Ab dupilumab analogue. To improve functionality, we conducted affinity maturation by sequential mutagenesis on CDRs of Abs^21,41^. The finally generated 4 R34.1.19 Ab specifically bound to IL-4Rα with higher affinity (*K*_D_ ≈ 178 pM) and more efficiently blocked the IL-4Rα-mediated signaling as a dual antagonist of both IL-4 and IL-13 cytokines than the parental Abs with comparable functionality to that of dupilumab analogue.

Epitope mapping by alanine scanning mutagenesis, combined with IL-4 binding competition assay, revealed that 4R34.1.19 Ab recognizes mainly the ligand (IL-4/IL-13) binding sites on IL-4Rα (Fig. 4), demonstrating that they orthosterically block the ligand binding to IL-4Rα. The binding epitopes of 4R34.1.19 were different from those of dupilumab analogue. Though dupilumab analogue competed with IL-4 for IL-4Rα binding, it substantially bound to most of the tested IL-4Rα alanine mutants, suggesting that dupilumab analogue mainly bind to the neighbouring residues around the ligand binding sites on IL-4Rα to sterically hinder access of the large IL-4 ligand to IL-4Rα^42^. Intriguingly, the relative binding tendency to each IL-4Rα alanine mutant was slightly different among 4R34, 4R34.1, and 4R34.1.19 (Fig. 4c), indicating that epitope drift occurs during the engineering of five CDRs in addition to the affinity improvement. For example, compared with the parental Abs (4R34 and 4R34.1), 4R34.1.19 rather recovered the binding affinity for IL-4Rα alanine mutants at D92 and V93, suggesting that the stronger interactions of 4R34.1.19 with other ligand binding sites lead to a more potent IL-4Rα antagonistic activity than the parental Abs.

In addition to binding affinity, biological activity of a given Ab often depends on the specific epitope on antigen^43^. Although 4R34.1.19 showed approximately 20-fold lower binding affinity with IL-4Rα than dupilumab analogue, mainly because of the faster dissociation rates than that of dupilumab analogue (Table 1), it showed comparable IL-4Rα antagonistic activity to that of dupilumab analogue in the reporter cells (Fig. 3d,e) and in IL-4-stimulated T cell proliferation and T_H_2 differentiation assay (Fig. 5). We speculated that the different binding epitopes between 4R34.1.19 and dupilumab analogue explains the comparable functional activity despite the difference in the affinities. Because Ab 4R34.1.19 bound to IL-4Rα at more identical sites with IL-4 compared with dupilumab analogue (Fig. 4c), 4R34.1.19 could induce IL-4Rα internalization, as the ligand IL-4 binding does^44,45^, resulting in IL-4Rα downregulation to abolish the function in addition to the ligand blocking. Further study is necessary to definitively account for this mechanism.

Because IL-4-mediated CD4^+^ T cell proliferation and its differentiation into T_H_2 differentiation are the upstream events triggering the downstream inflammatory cascade in allergic diseases^3^, blocking capability of the upstream events is critical in therapeutic anti-IL-4Rα Abs. 4R34.1.19 Ab efficiently inhibited IL-4-dependent proliferation of naïve T cells among PBMCs of two healthy donors, the activity of which was comparable to that of dupilumab analogue (Fig. 5a). Importantly, 4R34.1.19 Ab showed equivalent activity to that of dupilumab analogue in suppressive of the differentiation of patient-derived naïve CD4^+^ T cells into the T_H_2 cells (Fig. 5b,c). These data indicate the potential of 4R34.19 as a therapeutic Ab for the treatment of allergic diseases. Unfortunately, 4R34.1.19 did not cross-react with mouse IL-4Rα, similar to dupilumab (Patent US 7,605,237 B2), most likely because of the low sequence identity (~50%) in the ectodomain of IL-4Rα between human and mouse. Thus, we could not evaluate the *in vivo* efficacy.

In conclusion, we successfully generated a high affinity Ab, 4R34.1.19, directed against human IL-4Rα using yeast display technology. 4R34.1.19 showed comparable IL-4Rα-antagonistic functional activity in *ex vivo* assays with human T cells from healthy donors and asthmatic patients to that of dupilumab analogue. Although 4R34.1.19 has lower affinity with IL-4Rα than dupilumab analogue, it was distinguished from dupilumab analogue in the binding epitopes, indicating that both affinity and epitope are the critical factors to determine the biological activity of anti-IL-4Rα Abs. These different properties of 4R34.1.19 may lead to differential therapeutic efficacy from dupilumab analogue. Further, as seen with clinical utility of several Abs against tumor necrosis factor α^46^, alternative Abs are required for the same target because anti-drug Ab may be produced by repeated administration of the same therapeutic Ab for the chronic diseases. Finally, 4R34.1.19 is a potential candidate for further testing in human clinical trials for allergic diseases.

## Methods

### Protein expression and purification

The extracellular domain of human IL-4Rα genes (residues 26–232) were prepared by DNA synthesis (Bioneer Inc., Deajeon, Korea). The wild-type IL-4Rα and its alanine mutants (Y38A, L67A, L68A, D92A, V93A, S95A, D97A, and Y208A) were subcloned in frame into pSecTag2A vector to be expressed in the C-terminal 6× His-fused form. For hIL-4-mFc expression, the cDNA plasmids carrying human IL-4 (residues 1–153) genes (Sino biological Inc.,HG-11846-CM) were subcloned in frame into pcDNA3.4 vector to be expressed in the C-terminal mouse immunoglobulin Fc (hinge-CH2-CH3) fused form^30^. The variable regions (VH and VL) of isolated Fab clones and dupilumab were reformatted into the human IgG1 isotype through subcloning of respective VH and VL genes into the modified pcDNA 3.4 heavy chain vector (Invitrogen) carrying the human IgG1 constant domain and the pcDNA 3.4 light chain vector carrying human kappa constant domain, respectively, as described previously^16,47^. The plasmids encoding fusion proteins and Abs were transiently transfected into HEK293F cell cultures in Freestyle 293F media (Invitrogen, 12338018) following the standard protocol^47^. Wild-type IL-4Rα and IL-4Rα alanine mutants were purified using Ni-NTA resin (GE Healthcare, 17531801). Abs and hIL4-mFc protein were purified using Protein-A agarose chromatography column (CaptivA, CA-PRI-0100)^48^. Protein concentrations were determined using a Bicinchoninic Acid (BCA) kit (Pierce, 23225) by measuring the absorbance at 562 nm. To prepare Ab screening antigen probe, the purified 6× His-fused IL-4Rα were biotinylated using EZ-Link Sulfo-NHS-Biotin (Thermo Scientific, 21217) in accordance with the manufacturer’s instructions.

### Screening of yeast human synthetic Fab library against IL-4Rα

Yeast strains and media composition have been previously described^16,30^. The synthetic human Fab library displayed on the surface of yeast diploid cells with diversity of more than 5 × 10^9^ was used^16^. The library screening was performed by one round of MACS with 1 μM biotinylated IL-4Rα, followed by three rounds of FACS using a FACS Aria III (BD Biosciences) against biotinylated IL-4Rα (0.5 μM in round 1, 0.1 μM in round 2, and 50 nM in round 3). The cell surface expression and binding level of biotinylated IL-4Rα of the library were determined by indirect double immunofluorescence labelling of a light chain C-terminal Flag tag with anti-Flag Ab (Sigma, F3165, dilution 1:200) with Alexa488-labeled anti-mouse goat Ab (Invitrogen, A28175, dilution 1:600) and streptavidin-conjugated R-phycoerythrin (SA-PE) (Invitrogen, S866, dilution 1:600), respectively. Typically, the top 0.5–1% of target-binding cells were sorted. The sorted yeast cells were plated on the selective medium and individual clones were randomly chosen. The Fab DNA sequence was identified by yeast colony polymerase chain reaction (PCR)^16^.

### Affinity maturation of Abs

Affinity maturation of Abs by CDR mutagenesis was conducted in the scFab format containing a linker with 63 amino acids between the light chain and the heavy chain^27^. In the affinity maturation of 4R34, the targeted residues in VH-CDR3 (residues 95–98), VH-CDR2 (residues 50, 52–57), and VL-CDR3 (residues 89, 90, 93, 95a) were randomized using hand-mixed spiked oligonucleotides^25,26^, as described in the text. In the affinity maturation of 4R34.1, the targeted residues of VL-CDR1 (residues 27–32) and VH-CDR1 (residues 31–35) were randomized with a degenerate codon NNK^26^. For library construction, each amplified scFab gene library (12 μg) and linearized yeast surface display vector (4 μg) was co-transformed ten times into Saccharomyces cerevisiae EBY100 strain by a homologous recombination technique using a Gene Pulser II (BioRad)^16,26^. The diversity of libraries were determined by plating serial 10-fold dilutions of the transformed cells onto selective agar plates^30^. The library was screened using the FACS Aria III against biotinylated IL-4Rα in the presence of excess amount of non-biotinylated IL-4Rα as a competitor under equilibrium (low antigen concentration) and/or kinetic (long dissociation time) labelling conditions^28,29^, as specified in the text. During FACS, the cell surface expression and antigen binding levels of the scFab library were monitored by indirect double immunofluorescence labelling of the CH1 C-terminal c-myc tag (anti-c-myc mouse Ab (9E10), 13–2500, dilution 1:200)/Alexa488-labeled anti-mouse goat Ab (Invitrogen, dilution 1:600) and the IL-4Rα protein (biotinylated antigen/SA-PE). Typically, the top 0.1–0.5% of target-binding cells were sorted. After the final sorting, individual clones were analysed as described above.

### Binding analysis by ELISA

Binding specificity of isolated Abs to the plate-coated antigens (50 ng/well of IL-4Rα or GST) was analysed by indirect ELISA, as previously described^49^. For epitope mapping of anti-IL-4Rα Abs, wild-type IL-4Rα or its alanine mutants were coated to 96-well plates (5 ng/well). The wells were subsequently blocked with a blocking PBST buffer (Phosphate buffer saline (PBS), pH 7.4, 0.1% (v/v) Tween-20, 4%(w/v) Bovine serum albumin (BSA)). After washing thrice with PBST, wells were treated with 5 nM hIL-4 mFc or 2.5 nM–100 pM Abs in blocking buffer at 25 °C for 1 h. The plates were washed and developed as previously described^49^. The bound proteins were detected by adding horse radish peroxidase (HRP)-conjugated anti-human Fc Ab (Invitrogen, 628420, dilution 1:8000) or HRP-conjugated anti-mouse Fc Ab (Abcam, ab6789 dilution 1:4000). The data were presented as relative Ab binding to each alanine mutant after normalization of the Ab binding against wild-type IL-4Rα.

For competitive ELISA, various concentrations of anti-IL-4Rα Abs (20, 100, and 500 nM) were added for competitive binding of 6× His-fused IL-4Rα protein (50 nM or 10 nM) to the plate-coated hIL-4-mFc (50 ng/well) at 25 °C for 1 h. After washing thrice, bound 6× His-fused IL-4Rα protein was detected with HRP-conjugated anti-His Ab (Sigma, A7058, dilution 1:2000). The binding data were presented as the percentage of bound 6× His-fused IL-4Rα to hIL-4-mFc without competition.

### Non-specificity ELISA

The four different antigens of dsDNA (Sigma, 438545-06-3), cardiolipin (Sigma, C0563), hemocyanin (Sigma, H8283), and insulin (Sigma, I9278) were coated on ELISA plates following the previous conditions^34^: dsDNA (30 ng/well), cardiolipin (1.5 µg/well), hemocyanin (150 ng/well), and insulin (150 ng/well). The subsequent ELISA procedures were performed as described above.

### Size-exclusion chromatography

SEC analysis of purified antibodies was performed on the Agilent 1100 high performance liquid chromatography system using a superdex^TM^200 10/300GC (10 mm × 300 mm, GE Healthcare) size-exclusion column with a mobile phase of PBS buffer (pH 7.4) at a flow rate 0.75 ml min^−1^.

### Determination of Ab affinity by bio-layer interferometry

For anti-IL-4Rα Abs, we determined binding kinetic parameters for IL-4Rα using OctetRED96 instrument (ForteBio). All kinetic experiments were conducted at 25 °C with orbital shaking at 1000 rpm in 200 µL in 96-well black flat-bottom plates (VWR International, 82050-784). Each purified Ab was diluted to 1 μg/mL in kinetics buffer (PBS, pH 7.4, containing 0.02% (v/v) Tween 20) and directly immobilized onto anti-human IgG Fc capture (AHC) biosensors (ForteBio) at approximately 0.6 to 0.8 nm response. After an equilibration step of 300 s, the binding isotherms were monitored by exposing separate sensors simultaneously to different concentrations of IL-4Rα. The association of the antigen was measured for 300 s, followed by a dissociation step for 600 s to 900 s. For all experiments, an empty reference sensor without IL-4Rα antigen was used to account for nonspecific binding of analyte to the sensor. Association and dissociation rate constants were calculated by fitting to sensograms using the 1:1 binding model included in the Octet Data Analysis software 11.0 (ForteBio).

### HEK-Blue^TM^ IL-4/IL-13 reporter assay

HEK-Blue^TM^ IL-4/IL-13 cells were purchased from InvivoGen (hkb-il413)^19^. The blocking activity of anti-IL-4Rα Abs for IL-4/IL-13 signaling was assayed according to the manufacturer’s instructions. Briefly, the reporter cells (3 × 10^5^) cultured in a well of flat-bottom 96-well plates were added with recombinant human IL (rhIL)-4 or rhIL-13 (Peprotech, 200-04/200-13) and anti-IL-4Rα Abs, as specified in the Figure legend. After 24 h, 20 μL of the culture supernatant was mixed with 180 μL of resuspended substrate (QUANTI-Blue™, rep-qb1) in a well of flat-bottom 96-well plates. The reaction was incubated for 1 h and then the absorbance was read at 620 nm using a Cytation 3 imaging multi-mode reader (Biotek).

### Binding specificity to cell surface-expressed IL-4Rα

IL-4Rα-expressing THP-1 and IL-4Rα-deficient Molt-4 cells were harvested, and the cells were treated with 10 μg of purified IgG1 (Kappa from human myeloma plasma, Sigma-Aldrich, I5154) in PBS (pH 7.4) to block the FcγR binding of anti-IL-4Rα Abs at 4 °C. After 30 min, indicated concentrations of anti-IL-4Rα Abs was added and incubated for 1 h at 4 °C. The surface-bound anti-IL-4Rα Abs were stained with fluorescein isothiocyanate-conjugated anti-human Fcγ-specific F(ab’)_2_ monoclonal Ab for 30 min at 4 °C. After washing with 1 mL ice-cold PBS buffer, the cells were analysed by FACSCalibur (Becton-Dickinson) and FlowJo V10 software (Tree Star, San Carlos).

### Human PBMCs

All blood samples from asthmatic patients (over 25 years old) were acquired according to an approved protocol of Institutional Review Board (IRB) of Ajou University Hospital (approval ID: AJIRB-GEN-SMP-13-108) with written informed consent obtained from each patient. There were no restrictions on subtype, smoking status, and race. PBMCs from healthy donors (over 25 years old) were collected through an approved protocol by the Institutional Review Board (IRB) of Ajou University (approval ID: 201602-HM-001-01). All experiments were performed according to the approved protocols. PBMCs were isolated from five healthy donors and asthmatic patients by the Ficoll-Paque (GE Healthcare, 17-1440-02) density centrifugation method. These cells were then partially depleted of monocytes by plastic adherence, and non-adherent cells were suspended in X-VIVO15 media (Lonza, 04-744Q) supplemented with 2% (v/v) human AB serum (Sigma-Aldrich, H4522) for further experiments.

### Inhibition of IL-4-dependent T cell proliferation

PBMCs isolated from healthy donors were suspended in X-VIVO15 media containing 2% human AB serum, 10 μg/mL phytohemagglutinin P (PHA-P) (Sigma-Aldrich, L8754) and 20 IU/mL rhIL-2 (Thermo Fisher Scientific, PHC0026) and cultured for 3 days. Activated PBMCs (2 × 10^4^) by PHA-P stimulation were washed and plated in 96-well plates containing diluted anti-IL-4Rα Abs and rhIL-4 at indicated concentrations in Figure legend. Cells were incubated at 37 °C for 72 h and Cell-Titer Glo (CTG) reagent (Promega, G7570) was dispensed at 50 µL/well. The plates were incubated in the dark for 10 min and luminescence was detected using a cytation 3 cell imaging multi-mode reader (Biotech, 1 s exposure, 1x binning). The results are presented as the percentage of cells treated with anti-IL-4Rα Abs versus the PBS control.

### Inhibition of IL-4-dependent TH2 cell differentiation

PBMCs isolated from healthy donors or asthmatic patients were stained with Abs against CD4 and CD45RO. Naïve CD4^+^CD45RO^− ^T cells (5 × 10^4^ cells) were purified by FACS Aria III. The purified naïve CD4^+^CD45RO^− ^T cells were cultured for 7 days with stimulation of anti-CD3 Ab/anti-CD28 Ab coated sulfate latex bead (1:1 ratio) in the presence of T_H_2 differentiation conditions (5 μg/mL anti-interferon (IFN)-γ Ab, 5 ng/mL IL-2 and 10 ng/mL IL-4) and anti-IL-4Rα Abs^39^. Then, the cells were transferred to ELISPOT plate to measure the number of IL-4-secreting T_H_2 cells according to the manufacturer’s instructions (MabTech, 3410-4APW-2). Briefly, cultured T cells (1 × 10^5^ cells) were harvested and re-stimulated with anti-CD3 Ab/anti-CD28 Ab coated sulfate latex bead in ELISPOT plate coated with anti-IL-4 capture Ab. After 48 h incubation at 37 °C, cells were aspirated, and plates were washed with PBS. Biotin-conjugated anti-IL-4 Ab was added to the plates and incubated at room temperature for 2 h. Following additional washing steps, the plates were developed using Avidin alkaline phosphatase (MabTech) at 25 °C for 1 h, washed again with PBS and incubated with BCIP/NBT-plus substrate for 30 min at 25 °C in the dark. Substrate reaction was stopped by washing the plates with tap water. Once the membranes had dried, they were digitally scanned, and spot counts were determined by the ImmunoSpot Series 45 Micro ELISPOT Analyzer.

### Statistical analysis

Data are presented as the mean ± SD for representative data from at least three independent experiments, unless otherwise specified. One-way or two-way ANOVA with the Newman-Keuls post hoc test was used to evaluate the significance of differences between the indicated two or more groups using GraphPad Prism 5 software (GraphPad). A *P* value less than 0.05 was considered statistically significant.

## Supplementary information

Supplementary data

## Data Availability

All data in this study are available within the article or from the authors on request.
