# Supplementary material for: Engineering of anti-human interleukin-4 receptor alpha antibodies with potent antagonistic activity
Source: Sci Rep. 2019 May 23;9:7772. doi: 10.1038/s41598-019-44253-9 (PMC6533264; doi:10.1038/s41598-019-44253-9)
Supplement: Supplementary file 1 — Supplementary data [file 41598_2019_44253_MOESM1_ESM.pdf]

Engineering of anti-human interleukin-4 receptor alpha antibodies with potent antagonistic activity

Jung-Eun Kim, Keunok Jung, Jeong-Ah Kim, Seung-Hyun Kim, Hae-Sim Park and Yong-Sung Kim

Inventory of Supplementary Information

Supplementary Figure 1
Supplementary Figure 2
Supplementary Figure 3
Supplementary Figure 4
Supplementary Figure 5

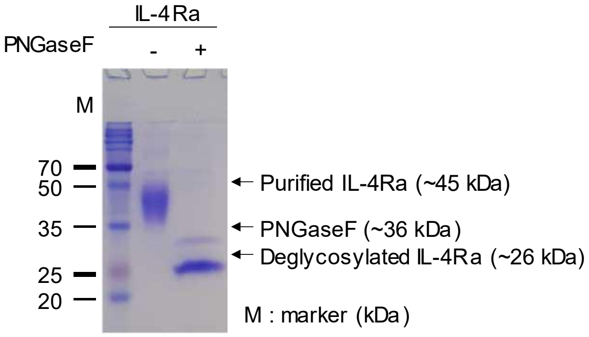

Supplementary Figure 1. Expression and purification of hIL-4Rα protein. The purified human IL-4Rα proteins (10 μg) from HEK293F cultures were treated with PNGase F (2500 units, NEB) at 37 °C for 24 h and then were analyzed by SDS-PAGE under the non-reducing condition. The arrows indicate the eluted position of the proteins. The smear band and size discrepancy of purified IL-4Rα proteins were because of the heterogeneous glycosylation. M indicates marker.

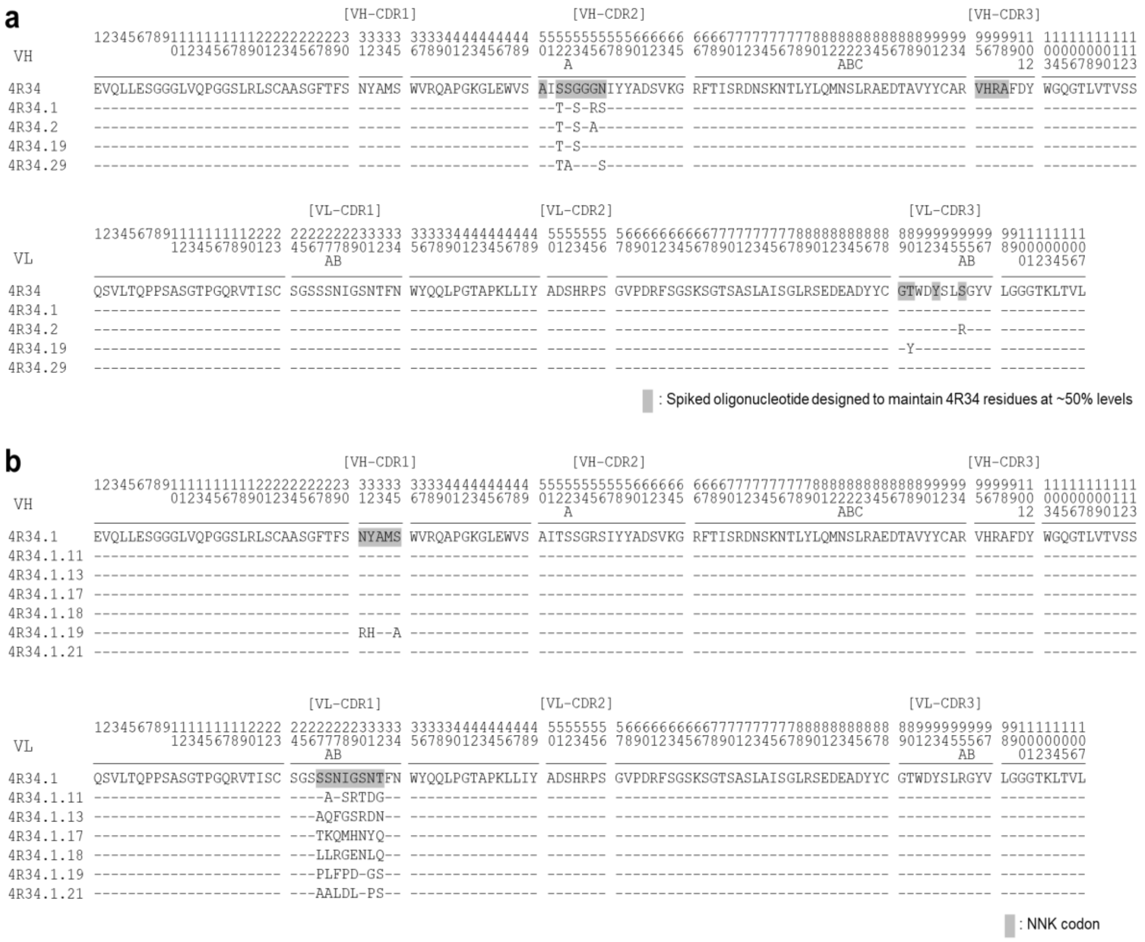

Supplementary Figure 2. Sequence Alignment of isolated clones from first and second affinity maturation library. The VH and VL sequences of isolated clones are compared with those of the parent antibodies (Abs). Dash indicates the identical sequence. The randomized residues in the parent Abs are shaded in grey. The residues are numbered according to the Kabat numbering scheme.

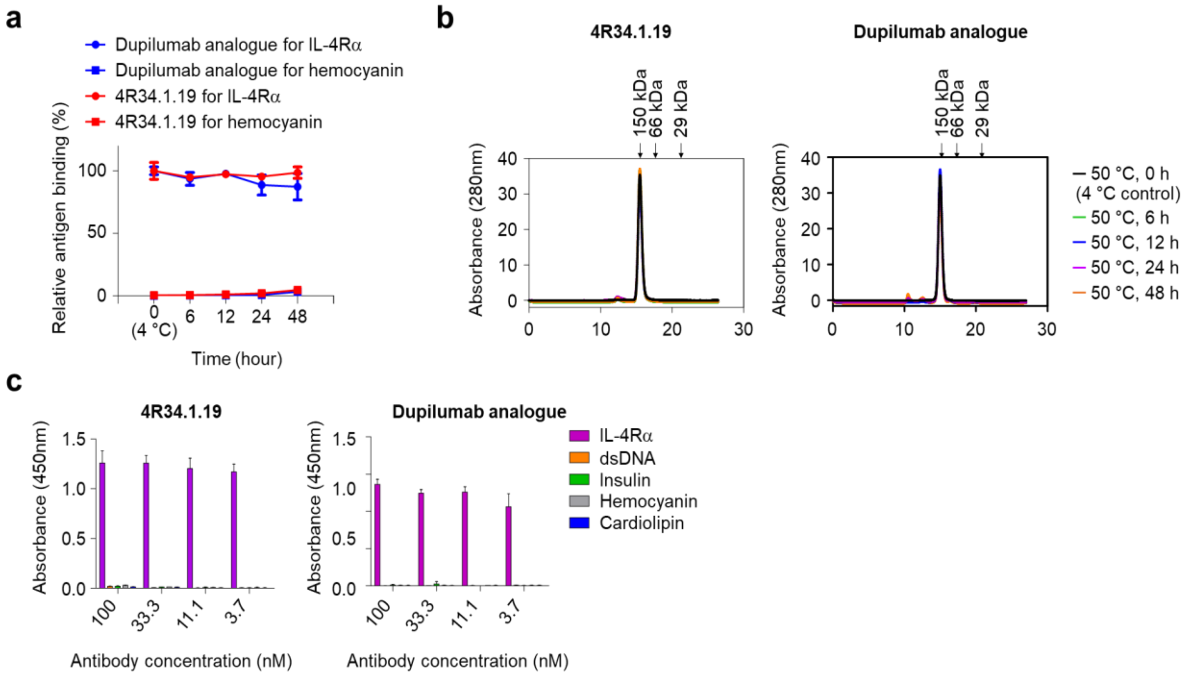

Supplementary Figure 3. 4R34.1.19 possesses favorable developability comparable to that of dupilumab analogue. (**a**,**b**) Thermal stability of 4R34.1.19 and dupilumab analogue was assessed by ELISA for the selective IL-4Rα antigen binding (**a**) and size exclusion chromatography (**b**) after incubating the Abs (2 mg/ml) at 50 °C for the indicated periods. In (**a**), the binding activity (%) was relative to the initial binding of Ab stored at 4 °C. Error bars represent the mean ± s.d. (n = 3). In (**b**), size exclusion chromatogram of Abs (10 μl of 2 mg/ml) was monitored at 280 nm. Ab stored at 4 °C was included as a control. Two independent analyses were performed with the same results. The arrows indicate the elution positions of molecular weight standards. (**c**) Evaluation of non-specific binding activities of 4R34.1.19 and dupilumab analogue Abs to four different antigens (dsDNA, insulin, hemocyanin, and cardiolipin), determined by ELISA.

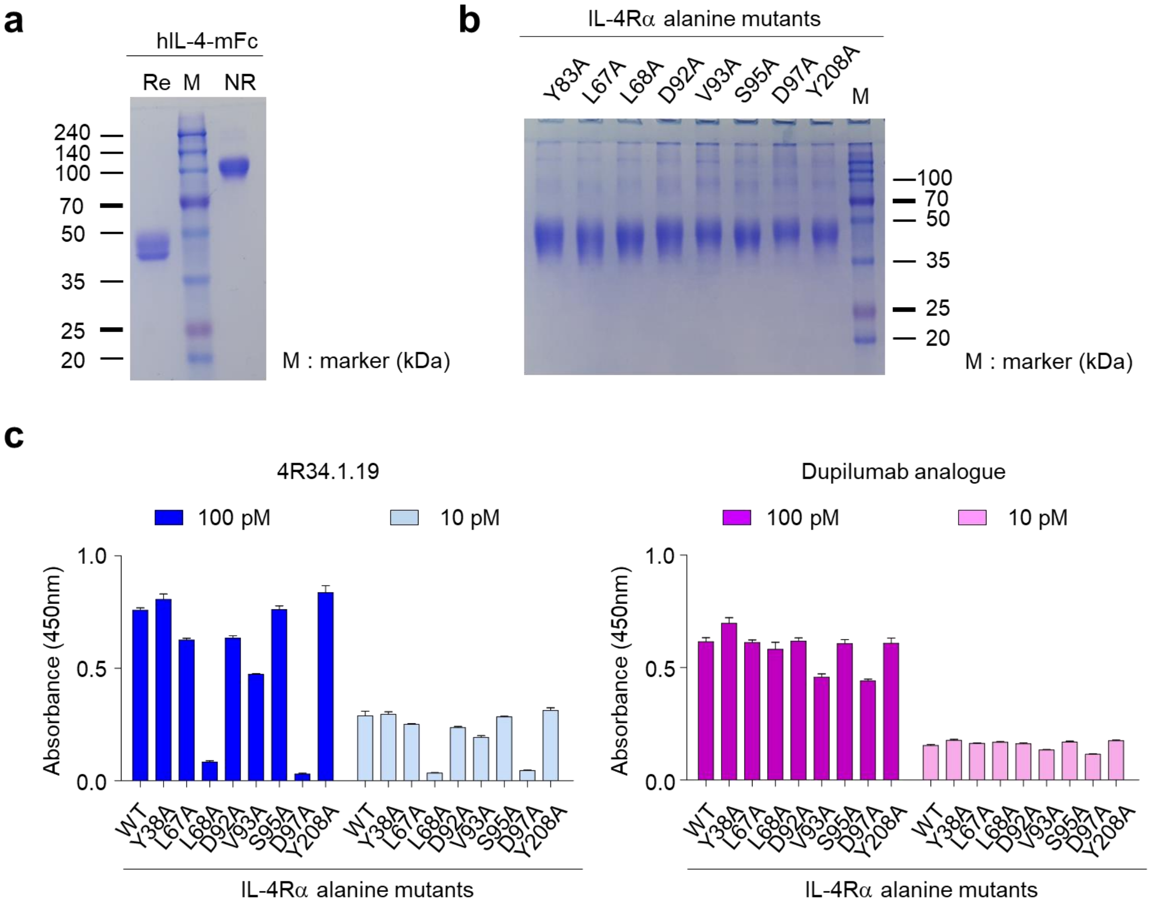

Supplementary Figure 4. **Epitope mapping of anti-IL-4Rα Abs.** (**a**,**b**) Purification of hIL-4-mFc protein and IL-4Rα alanine mutants. The purified hIL-4-mFc proteins (a) and IL-4Rα mutants with the indicated alanine substitution (b) from HEK293F cultures are separated on 10 % SDS-PAGE and then stained with Coomassie brilliant Blue. The hIL-4-mFc proteins (5 μg) are analyzed under reducing (R) and non-reducing (NR) conditions. The IL-4Rα alanine mutants (5 μg) is analyzed under the non-reducing condition and the smear bands are because of the heterogeneous glycosylation. The marker lane was cropped from different parts of the same gel. (**c**) Concentration-dependent binding ELISA of 4R34.1.19 and dupilumab analogue (10 and 100 pM) for wild-type IL-4Rα and the indicated alanine mutants. Data are represented as mean ± SD (n = 3). The percent relative binding of Abs at 100 pM is shown in Fig. 4c.

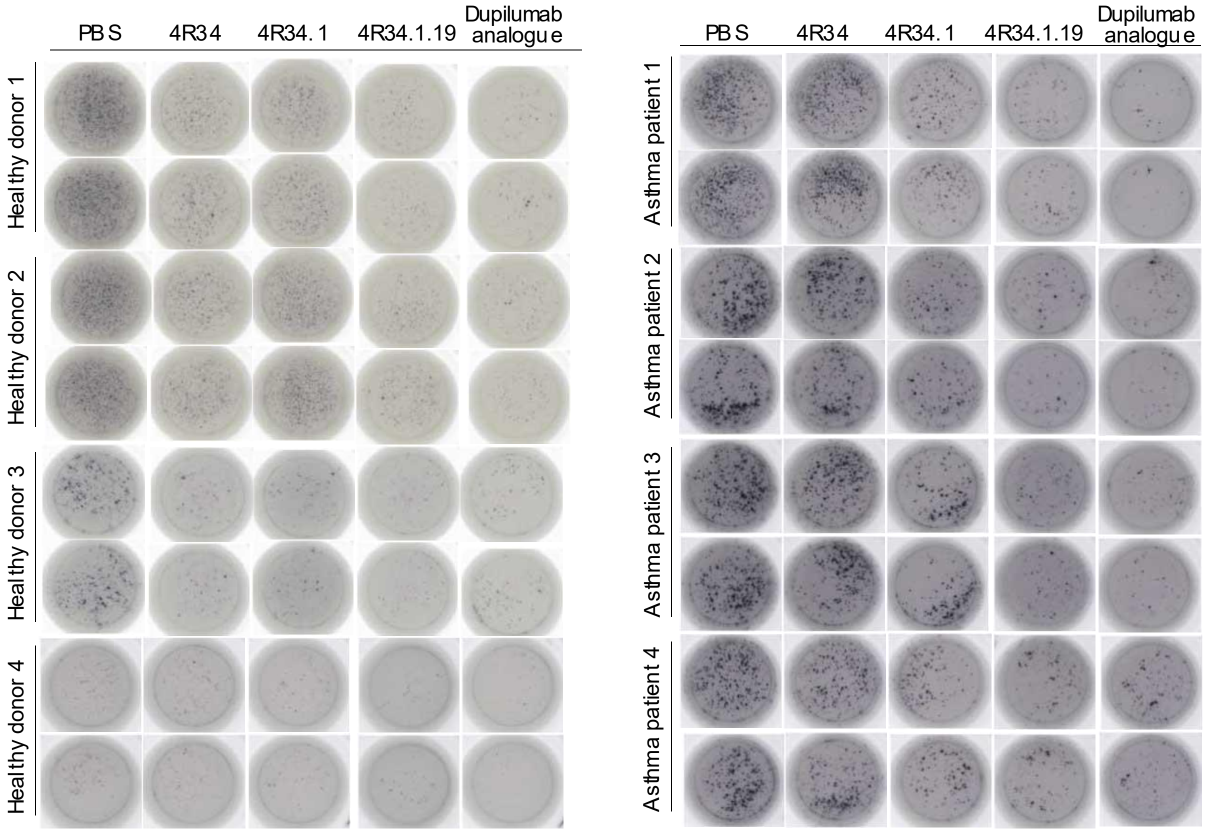

Supplementary Figure 5. **Inhibitory effects of anti-IL-4Rα Abs on the T_H2 differentiation of IL-4-stimulated naïve $CD4^+$ T cells**. Images showing the inhibitory effect of anti-IL-4Rα Abs on the T_H2 differentiation of naïve $CD4^+CD45RO^-$T cells from healthy donors (n=4) or asthmatic patients (n=4), determined by ELISPOT assay. Quantification of the number of IL-4-producing T cells is shown in Fig. 5b.
